# Spinal Cord Ischemia Following Endovascular Repair of Infrarenal Abdominal Aortic Aneurysm

**DOI:** 10.3400/avd.cr.20-00061

**Published:** 2020-09-25

**Authors:** Shingo Nakai, Tetsuro Uchida, Yoshinori Kuroda, Atsushi Yamashita, Eiichi Oba, Kimihiro Kobayashi, Tomonori Ochiai, Mitsuaki Sadahiro

**Affiliations:** 1 Second Department of Surgery, Yamagata University Faculty of Medicine

**Keywords:** spinal cord ischemia, paraplegia, EVAR

## Abstract

Spinal cord injury (SCI) following endovascular aortic repair (EVAR) for an abdominal aortic aneurysm (AAA) is a rare but serious complication. Case 1 presented with ruptured AAA and shock and underwent emergency EVAR. The patient developed incomplete paraplegia 2 days following EVAR. Case 2, diagnosed with impending rupture of AAA with extremely shaggy aorta, was treated with emergency EVAR. The patient was diagnosed with complete paraplegia soon after EVAR. Case 3 underwent elective EVAR and developed delayed paraplegia 2 weeks later. In EVAR, the etiology of SCI leading to paraplegia is often multifactorial. Surgeons must consider the possibility of SCI-induced paraplegia.

## Introduction

Spinal cord injury (SCI) following infrarenal abdominal aortic aneurysm (AAA) repair is an unusual but serious complication that impairs activities of daily living. Following endovascular aortic repair (EVAR), post-surgical paraplegia and paraparesis are less common than open surgery. Since 2010, 320 patients underwent EVAR for AAA with/without iliac arterial aneurysm in our hospital. Of these 320 patients, 3 (0.009%) experienced paraplegia. Herein, we report these 3 cases of SCI-related paraplegia following EVAR for AAA and consider the underlying pathophysiology.

## Case Report

### Case 1

**Figure 1:**
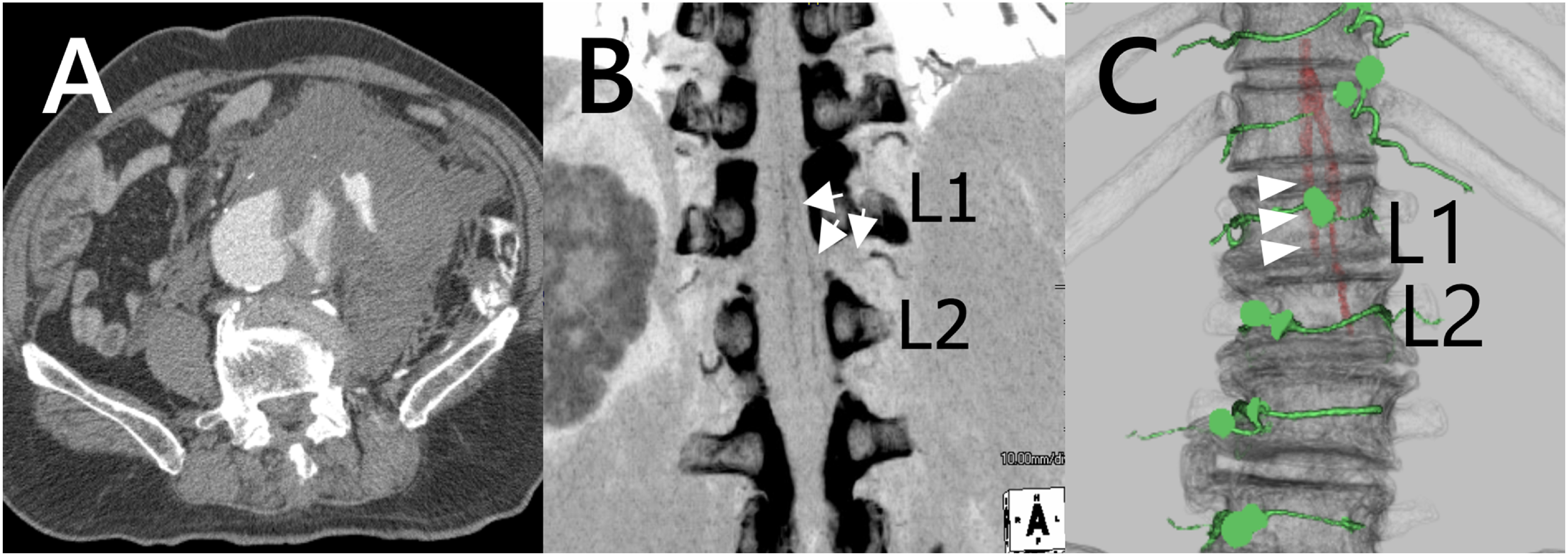
Fig. 1 (**A**) Preoperative computed tomography reveals a 51 mm serial left common iliac artery aneurysm from the abdominal aortic aneurysm and a retroperitoneal hematoma. (**B**) Preoperative contrast-enhanced computed tomography showing the Adamkiewicz artery originating at the L1–L2 level (white arrows). (**C**) Three-dimensional computed tomographic reconstruction: The Adamkiewicz artery is indicated by white arrow heads.

A 79-year-old man with a 66 mm ruptured AAA was admitted to our hospital in shock. Computed tomography (CT) revealed a 51 mm serial left common iliac artery (CIA) aneurysm from the AAA, retroperitoneal hematoma, and evidence of active bleeding from the anterior wall of the AAA (**Fig. 1A**). EVAR was conducted as an emergency surgery. Before EVAR, an intra-aortic balloon was inserted and inflated at the descending aorta for hemostasis. Initially, the left internal iliac artery (IIA) was occluded by using embolization coils (Tornado® Embolization Coil, Cook Inc., Bloomington, IN, USA). A bifurcated endovascular graft (Gore Excluder Endoprosthesis, W. L. Gore & Associates Inc., Flagstaff, AZ, USA) was deployed at the infrarenal lesion to land distally in the right CIA and external iliac artery (EIA) with limb extension. The patient’s blood pressure increased immediately after completion of EVAR, although more than 4 h had passed since the onset of the AAA rupture. The operation time was 115 min.

On the second postoperative day, the patient was found to have developed left lower limb incomplete paraplegia. Based on suspicion of SCI, the patient was treated with cerebrospinal fluid (CSF) drainage, high-dose steroid infusions, naloxone, and edaravone. Magnetic resonance imaging (MRI) identified spinal cord infarction at T11–L1. Retrospective analysis of preoperative CT scans revealed the origin of the Adamkiewicz artery at L1–L2 level (**Figs. 1B** and **1C**). This origin may have been unintentionally covered by the endograft. Based on patient records, the patient did not have any drop in his blood pressure below 80 mmHg in the postoperative period. Currently, the patient is receiving walking rehabilitation; his bladder incontinence has remained unchanged 43 months post-surgery.

### Case 2

**Figure 2:**
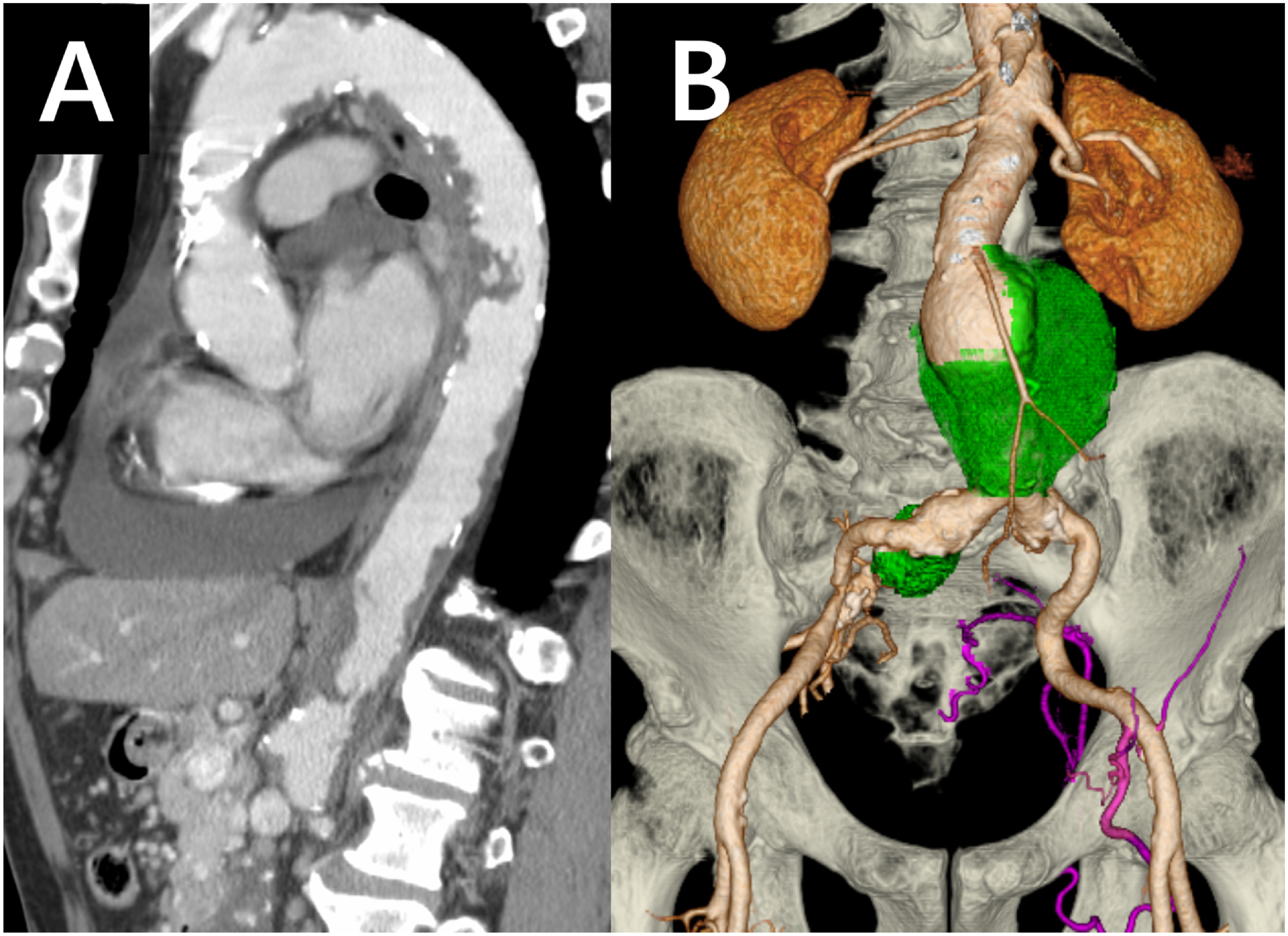
Fig. 2 (**A**) Contrast-enhanced computed tomography images conducted 4 years before the current presentation. The aorta has very severe atherosclerotic changes and mobile plaques. (**B**) Preoperative three-dimensional computed tomography in Case 3 shows a 56 mm infrarenal abdominal aortic aneurysm, a 26 mm right internal iliac artery (IIA) aneurysm, and collateral supply from the left femoral artery to the distal end of the occluded left IIA (pink color).

A 76-year-old man with AAA presenting with severe abdominal pain was found to have a 60 mm infrarenal AAA on non-contrast-enhanced CT. A contrast-enhanced CT conducted 4 years ago showed an extremely shaggy aorta (**Fig. 2A**). This patient was considered unsuitable for open surgery because of his poor general condition, severe renal dysfunction, and sarcopenia. Emergency EVAR was conducted to prevent AAA rupture. The right IIA was occluded by using embolization coils since the landing length of right CIA was short (15 mm) with a hostile neck and raised concerns regarding a type1b endoleak. This was followed by EVAR with Gore Excluder endoprosthesis. The distal landing sites were left CIA and right EIA with limb extension. The patient was diagnosed with complete paraplegia soon after EVAR. Although SCI was intensively managed, including CSF drainage, steroid infusions, and naloxone, the patient died 9 days following EVAR secondary to bowel necrosis.

### Case 3

A 77-year-old man with AAA underwent elective EVAR. Preoperative CT scan identified a 56 mm infrarenal AAA and a 26 mm right IIA aneurysm, with chronic total occlusion of left IIA, and collateral circulation from the left femoral artery (**Fig. 2B**). At first, the right IIA was embolized by using Tornado coils. Subsequently, EVAR was conducted using an Endurant stent graft system (Medtronic, Inc., Santa Rosa, CA, USA). Distal legs of the endograft were placed in the right EIA and left CIA. Four days after EVAR, the patient complained of right lower limb paresis. Brain MRI and CT scans did not identify any abnormal findings. Initially, the patient appeared to recover well and was able to walk around the ward. However, on the 12th postoperative day, he developed right lower limb paralysis for the second time. Emergency spinal MRI confirmed subacute spinal cord infarction at T3–T6. He was treated medically without CSF drainage. Although his muscle power improved to the point that he could walk with a cane, the patient died of lung cancer 21 months later.

## Discussion

Spinal cord ischemia leading to paraplegia after AAA repair is a rare but serious complication. The incidence of SCI is 0.1%–0.2% following elective open AAA surgery and 1.4%–2.0%, following emergency surgery for ruptured AAA.^1)^

EVAR has brought about a major paradigm shift in the treatment of AAA. However, similar to open surgery, postoperative SCI has also been reported following EVAR. According to the Eurostar database, the incidence of SCI after EVAR was reported as 0.21%.^2)^ The cause of SCI after EVAR is not clearly understood, and various factors may contribute to its pathogenesis, such as prolonged aortic clamping, perioperative hypotension, atheromatous embolization, interruption of the great radicular artery (the Adamkiewicz artery), or collateral perfusion through pelvic circulation.^3)^ **Table 1** summarizes the possible causes of SCI in our cases.

**Table 1:** Table 1 Summary of three cases of paraplegia

	The possible risk factors for developing paraplegia	Onset and severity	Outcome
Case 1	Prolonged hypotension	POD 2 (subacute)	Alive
	Occlusion of the Adamkiewicz artery	incomplete	Walking with a cane
Case 2	Micro embolism due to manipulation in severely shaggy aorta	Immediately after EVAR (acute)	Died
		complete	No improvement
Case 3	Instability of pelvic circulation	POD 4 and POD 12 (delayed)	Alive
	(occlusion of Bilateral IIA, IMA, lumbar artery, etc.)	incomplete	Walking with a cane

IIA: internal iliac artery; IMA: inferior mesenteric artery; POD: postoperative day

In Case 1, perioperative hypotension secondary to hemorrhagic shock was considered to be the main cause of paraplegia. In ruptured AAA, rapid completion of EVAR is required to achieve adequate arterial blood pressure. The interval between the onset of hypotension and EVAR completion could be prolonged because of IIA coiling. Peppelenbosch et al. proposed emergency EVAR with aorto-uni-iliac stent graft system to avoid SCI by minimizing IIA ischemia.^4)^ The Adamkiewicz artery is not occluded in most EVAR cases, because its typical origin is at the lumbar as well as the thoracic vertebral levels.^5,6)^ However, the Adamkiewicz artery in Case 1 originated only from the L1–L2 level. Thus, the spinal cord perfusion through the Adamkiewicz artery may have decreased because of stent graft coverage of its origin.

Case 2 was known to have a shaggy aorta with severe atherosclerotic changes based on imaging. Therefore, intraoperative embolism, which decreased the spinal cord perfusion, was considered as pathogenesis of SCI. Frequent device insertion and removal from the shaggy aorta may have contributed to SCI.^7)^ Gentle catheterization is mandatory for preventing paraplegia due to embolism.

In Case 3, the apparent cause of delayed SCI is not clear. However, bilateral occlusion of IIA may have been a possible cause of paraplegia. Collateral pelvic circulation, such as through IIA and lumbar artery, is known to be important for spinal cord perfusion. One report mentions a lack of neurological deficits in cases with bilateral IIA interruption following open as well as endovascular surgery for AAA.^8)^ Bratby et al. reported a 3% incidence of SCI in patients who underwent bilateral IIA embolization before EVAR.^9)^ There is no reference to the timing of delayed onset of SCI in literature. Goldstein et al. reported a case of delayed SCI following the use of an aorto-uni-iliac device wherein the patient developed paraplegia 3 weeks following the procedure. The authors reported that secondary hypotension might induce further reduction in spinal cord perfusion and contribute to the development of delayed paraplegia.^10)^ The precise pathophysiology in Case 3 could not be determined.

## Conclusion

EVAR is widely accepted as a less-invasive alternative to open surgery, especially in high-risk patients. However, being aware of the possibility of postoperative SCI following EVAR is important. The main causes of SCI following EVAR are multifactorial and vary from patient to patient. Some of the factors include pre-existing complications, anatomical characteristics of spinal cord perfusion, urgency of the operation, and general patient condition before surgery. Surgeons conducting EVAR should always exercise caution and be aware of the possibility of SCI-induced paraplegia as a procedural complication.
